# Public Perspectives on Anti-Diabetic Drugs: Exploratory Analysis of Twitter Posts

**DOI:** 10.2196/24681

**Published:** 2021-01-26

**Authors:** Su Golder, Millie Bach, Karen O'Connor, Robert Gross, Sean Hennessy, Graciela Gonzalez Hernandez

**Affiliations:** 1 Department of Health Sciences University of York York United Kingdom; 2 Department of Biostatistics and Epidemiology Perelman School of Medicine University of Pennsylvania Pennsylvania, PA United States; 3 Center for Clinical Epidemiology and Biostatistics Perelman School of Medicine University of Pennsylvania Pennsylvania, PA United States

**Keywords:** diabetes, insulin, Twitter, social media, infodemiology, infoveillance, social listening, cost, rationing

## Abstract

**Background:**

Diabetes mellitus is a major global public health issue where self-management is critical to reducing disease burden. Social media has been a powerful tool to understand public perceptions. Public perception of the drugs used for the treatment of diabetes may be useful for orienting interventions to increase adherence.

**Objective:**

The aim of this study was to explore the public perceptions of anti-diabetic drugs through the analysis of health-related tweets mentioning such medications.

**Methods:**

This study uses an infoveillance social listening approach to monitor public discourse using Twitter data. We coded 4000 tweets from January 1, 2019 to October 1, 2019 containing key terms related to anti-diabetic drugs by using qualitative content analysis. Tweets were coded for whether they were truly about an anti-diabetic drug and whether they were health-related. Health-related tweets were further coded based on who was tweeting, which anti-diabetic drug was being tweeted about, and the content discussed in the tweet. The main outcome of the analysis was the themes identified by analyzing the content of health-related tweets on anti-diabetic drugs.

**Results:**

We identified 1664 health-related tweets on 33 anti-diabetic drugs. A quarter (415/1664) of the tweets were confirmed to have been from people with diabetes, 17.9% (298/1664) from people posting about someone else, and 2.7% (45/1664) from health care professionals. However, the role of the tweeter was unidentifiable in two-thirds of the tweets. We identified 13 themes, with the health consequences of the cost of anti-diabetic drugs being the most extensively discussed, followed by the efficacy and availability. We also identified issues that patients may conceal from health care professionals, such as purchasing medications from unofficial sources.

**Conclusions:**

This study uses an infoveillance approach using Twitter data to explore public perceptions related to anti-diabetic drugs. This analysis gives an insight into the real-life issues that an individual faces when taking anti-diabetic drugs, and such findings may be incorporated into health policies to improve compliance and efficacy. This study suggests that there is a fear of not having access to anti-diabetic drugs due to cost or physical availability and highlights the impact of the sacrifices made to access anti-diabetic drugs. Along with screening for diabetes-related health issues, health care professionals should also ask their patients about any non–health-related concerns regarding their anti-diabetic drugs. The positive tweets about dietary changes indicate that people with type 2 diabetes may be more open to self-management than what the health care professionals believe.

## Introduction

In 2016, 4.2 million diabetes-related deaths were reported worldwide [1], which makes diabetes the seventh leading cause of mortality [2]. For both type 1 and type 2 diabetes, treatment and management aim to achieve adequate glycemic control [3]. Medication nonadherence is reported to be high for insulin and even higher for noninsulin anti-diabetic drugs [4,5]. Patients’ beliefs about medications, such as whether they are perceived to be essential or whether they have side effects, can influence both adherence and self-management behaviors [6]. The odds of nonadherence is 3.4 times as high in those who believe that anti-diabetic drugs have serious side effects and 14.3 times as high in people who believe that diabetes treatment regimens are too complex [7].

Given social media’s ability to connect large numbers of people and thereby generate large volumes of data, it has become a novel area for health research and a powerful tool to understand public perceptions. This study uses a particular social media site, that is, Twitter. As a popular social media outlet, Twitter is both a microblogging site and a social networking platform [8]. Since its conception in 2006 [9], Twitter’s popularity has grown to a reported 330 million monthly active users in 2019 [10]. The utilization of Twitter as a data collection platform is increasing and it is the most commonly utilized social media platform within health research [11]. Sinnenberg et al [12] demonstrated that the number of health-related studies harnessing Twitter in 2015 was over 10 times higher than that in 2010, and their systematic review of 137 studies identified many ways in which Twitter data can be used. The most common Twitter analyses identified by the authors were content analyses, wherein the words, pictures, or sentiment of tweets are analyzed. The monitoring of vocabulary within tweets for pharmacovigilance purposes is an expanding area of research [13], while the exploration of tweets discussing perceptions of medications can help understand compliance and therapeutic decision making [14]. With regard to diabetes, studies have examined changing sentiments in Tweets on diabetes since the COVID-19 outbreak [15], and public perceptions have been examined on Twitter in detail for diseases such as Ebola virus disease [16] and cancer [17] and products such as e-cigarettes [18].

In this study, we sought to identify perceptions held by people discussing anti-diabetic drugs on Twitter. In particular, we sought to assess 3 questions: (1) Who discusses anti-diabetic drugs on Twitter? (2) Which anti-diabetic drugs are the most frequently discussed on Twitter? and (3) What are the most common health-related topics discussed on Twitter regarding anti-diabetic drugs?

## Methods

**Figure 1:**
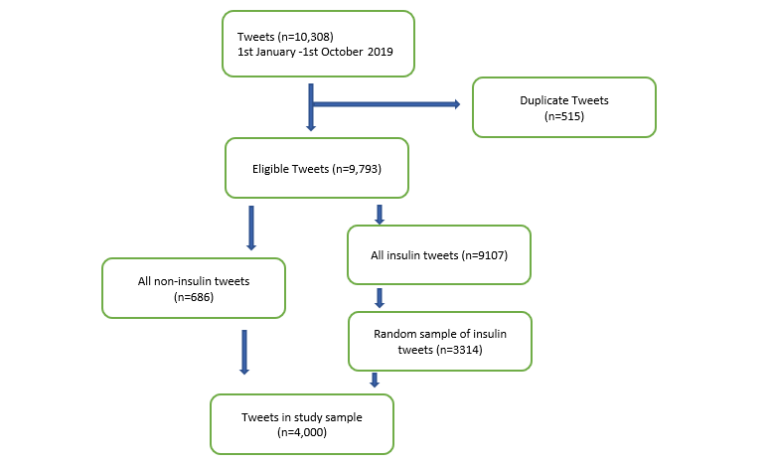
Flowchart summarizing the tweet selection process.

Publicly available tweets posted between January 1, 2019 and October 1, 2019 were retrieved by the University of Pennsylvania’s Health Language Processing Center [19] from a large publicly available data set curated by the Internet Archive. The Internet Archive is a nonprofit organization that builds digital libraries of internet sites and provides free access to the data to researchers. We removed retweets from the collection. We selected this time scale in order to account for any seasonal or newsworthy variations in the tweets posted. Search terms associated with anti-diabetic drugs, including generic names, brand names, and common misspellings (Multimedia Appendix 1) were used to retrieve 10,308 tweets (Figure 1). After removing 515 duplicates, 92.9% (9107/9793) of the medication-related tweets were found to be about insulin. We, therefore, constructed a purposive sample of all tweets about noninsulin anti-diabetic drugs (n=686) so as to not lose any potential valuable information and a random sample about insulin (n=3314).

Qualitative studies traditionally have small sample sizes [20], but social media analyses are associated with qualitative data on a quantitative scale [21]. Consequently, qualitative Twitter analyses often use a sample of tweets rather than the full sampling frame [22]: sample sizes range from a few hundred [23] to thousands of tweets [12]. Guided by previous research, we initially began with 4000 random tweets (4000/9793 or 40.8% of our total sample), with additional samples to be analyzed if code saturation and meaning saturation were not met. Code saturation can be defined as the point at which all codes have been identified, while meaning saturation is the point at which all codes are understood [24]. After coding all 4000 tweets, code saturation and meaning saturation appeared to have been met [24] and a further sample was not necessary. Codes are labels for assigning units of meaning [25]. In qualitative content analysis, the use of codes results in the generation of themes that can be used to interpret the meaning of the text [26]. Health-related tweets were coded based on the perception expressed in the tweet. This used the conventional content analysis inductive framework proposed by Hsieh and Shannon [27] to explore both the manifest and latent meanings of the tweets and ensured that the codes arose from the data itself rather than being predefined. An inductive approach was particularly useful as there is little theory on anti-diabetic drug perceptions discussed via Twitter on which to base any assumptions and there is no particular framework to work from. Inductive approaches on Twitter data are also commonplace in the scientific literature [16]. Initial codes were given to each tweet, and upon reflection of the whole data set, similar or linked codes were clustered into themes. Some similar themes were further combined to form subthemes under an overarching theme.

The themes identified at this stage formed the basis of the coding scheme. We created a manual containing the coding scheme and instructions with examples on how to correctly assign codes. We filtered the Internet Archive data set by matching the keywords list, which includes all anti-diabetic drugs and their variants in the tweets. Only tweets in English and those that were not retweets were retrieved. The output file created contains all tweets where a match was found and included the user ID, tweet ID, tweet text, data created, and the keyword that matched in separate columns in an Excel. The keyword column helped ascertain the drug mention; however, the themes were hand-coded from scratch [28].

**Figure 2:**
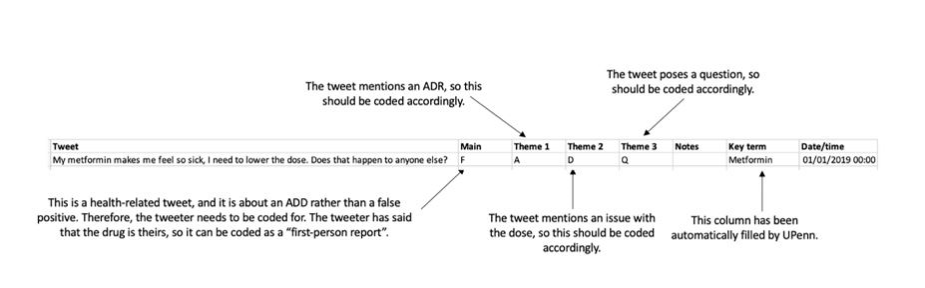
Coding example with a theoretical tweet. ADD: anti-diabetic drug; ADR: adverse drug reaction; UPenn: University of Pennsylvania.

Two researchers independently coded 231 tweets by using the coding scheme. A random sample of 231 tweets was found to be sufficient to measure agreement and to stimulate discussion on the coding scheme as all codes were represented multiple times in this sample size. Because the initial kappa coefficient was 0.67, disagreements were discussed, and the coding instructions adapted accordingly. A further 169 tweets were then coded independently by 2 reviewers, producing a satisfactory kappa score of 0.73 [29]. Each of the remaining tweets was then coded by one of the two researchers, with all codes checked by the other reviewer and any disagreements resolved by discussion. First, tweets were coded for whether they truly were anti-diabetic drug–related. Second, any anti-diabetic drug–related tweets were coded as either health-related or non–health-related. Health-related tweets were further coded. Tweeters were categorized as (1) those who used the drug themselves, (2) people who knew someone who takes the drug, (3) health care providers, or (4) unclear, that is, the relationship between the tweeter and the anti-diabetic drug was unclear. Figure 2 shows a theoretical tweet, which has been coded, to show how coding was performed.

The availability of social media data means that it is relatively easy to trace quotations back to the user; therefore, there is a risk of deductive disclosure [30]. This makes reporting direct quotations problematic. Subtle changes to tweets are at odds with the Twitter display requirements, which prevent the alteration of tweets [31]. We, therefore, undertook a descriptive approach through paraphrasing tweets and by only directly quoting commonly used terms so that they cannot be traced back to an individual tweet. All data used in this study were collected according to the Twitter terms of use and were publicly available at the time of collection and analysis. We have an institutional review board certificate of exemption from the University of Pennsylvania. Each theme was explored regardless of how often it occurred.

## Results

### Tweeter Description

The results of this study are based on the 1664 health-related tweets (Table 1). A quarter (415/1664, 24.9%) of the tweets were by patients with diabetes taking anti-diabetic drugs, or who had taken the anti-diabetic drug in the past or who might initiate the anti-diabetic drug in the future; 87 (21.1%) of these self-identified as having type 1 diabetes, 61 (14.6%) as having type 2 diabetes, 2 (0.5%) as having gestational diabetes, and 2 (0.5%) as having secondary diabetes. The type of diabetes could not be classified for two-thirds of the tweeters; 17.9% (298/1664) of the tweets were second-person accounts, often about a family member or a person in a news story, and 2.7% (45/1664) of the tweets were from health care professionals. We could not establish the relationship between the tweeter and the anti-diabetic drug for the remaining 54.4% (906/1664) of the tweets.

**Table 1:** Proportions of the types of tweets and tweeters.

Type of tweet/type of tweeter		Explanation	n (%), Value
**Irrelevant tweets (n=2336)**			
	Non–health-related	Tweets that mention an anti-diabetic drug but are not directly related to health, for example, jokes, advertisements.	1556 (66.6)^a^
	Not a drug	Key term is used but is not in reference to a drug, for example, using the term “insulin” to mean the endogenous hormone rather than the exogenous anti-diabetic drug.	693 (29.6)
	Not in English	The majority of the tweets were not in English.	7 (0.3)
	Not related to diabetes	Tweet refers to drug being used for a purpose other than diabetes.	80 (3.4)
**Health-related tweets (n=1664)**			
	First-person report	Tweet from a diabetic person—uses phrases like “my drug…”	415 (24.9)
	Second-person report	Tweets from someone who is not diabetic but is about a diabetic person—uses phrases like “my daughter’s drug…”	298 (17.9)
	Health care professional	Tweet is from a health care professional—uses phrases like “my patient’s drug”	45 (2.7)
	Inconclusive	There is insufficient context to determine who is sending the tweet.	906 (54.4)

^a^Of these, 920 (59.1%) tweets were on cost.

### Anti-Diabetic Drugs Under Discussion

Tweets related to 33 anti-diabetic drugs across 11 drug classes were identified: insulin (1281 tweets), biguanides (194), SGLT2 inhibitors (102), DDP4 inhibitors (33), GLP1 agonists (97), sulfonylureas (11), thiazolidinediones (16), metformin (2), α-glucosidase inhibitors (1), meglitinides (1), and amylase analogues. People tweeted using both generic and brand names.

### Common Perceptions

**Figure 3:**
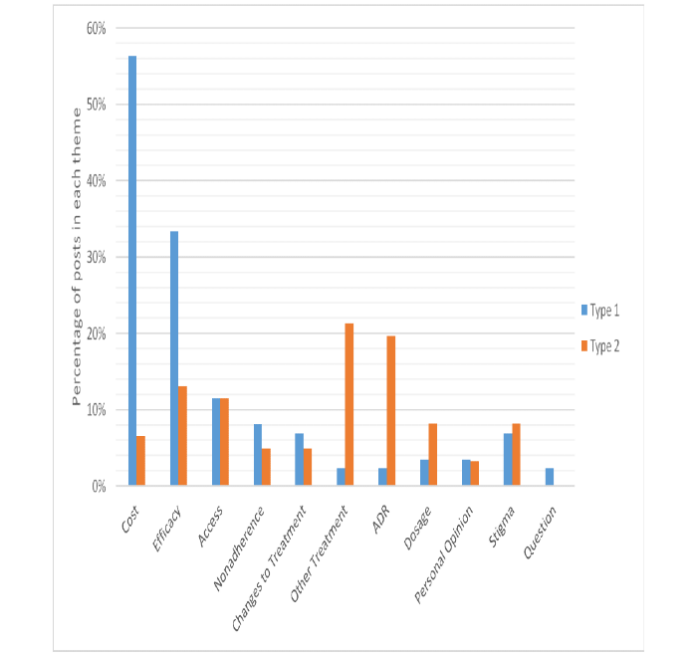
Tweet categories by people with type 1 and type 2 diabetes. ADR: adverse drug reaction.

We identified 13 themes (Table 2). In most cases, we could not determine if the tweet was about type 1 or type 2 diabetes. Cost and efficacy dominated type 1 diabetes posts and other treatments, and adverse drug reactions dominated type 2 diabetes tweets. Type 1 diabetes tweets were also more likely to discuss more than one topic (Figure 3).

**Table 2:** Themes of the health-related tweet categories (n=1664).

Theme	Explanation	Subthemes	n (%), Value
Cost	Tweet discusses the cost of an anti-diabetic drug in relation to health issues.	How much do anti-diabetic drugs cost? Attitudes toward cost, insurance problems, health consequences, social consequences, managing cost	669 (40.2)
Efficacy	Tweet discusses efficacy of the drug, both positive and negative. This includes tweets about the necessity of the drug and tweets that state that death will occur if the anti-diabetic drug is not taken.	Positive and negative	465 (27.9)
Information resource	Tweet provides information about the anti-diabetic drugs. These tweets reference research articles or clinical guidelines rather than someone’s belief about the anti-diabetic drugs.	Links and information summaries	371 (22.2)
Availability	Tweet discusses the availability of or access to anti-diabetic drugs.	Nationwide availability, personal availability, ensuring availability	158 (9.5)
Nonadherence	Tweet discusses someone not following the recommendation for taking the anti-diabetic drugs.	Taking too much, taking too little, consequences of nonadherence	124 (7.5)
Personal opinion	Tweet discusses a personal belief about anti-diabetic drugs.	Preferences, opinions of people without diabetes, opinions of people with diabetes	94 (5.6)
Other treatment options	Tweet compares an anti-diabetic drug to another management option for diabetes.	Other management options, effect on anti-diabetic drug, attitudes toward other treatments	54 (3.2)
Question	Tweet is being used to seek advice or to challenge others.	Advice from others, educational tool	41 (2.5)
Changes to treatment	Tweet discusses starting, stopping, or changing to another anti-diabetic drug.	Starting a medication, stopping a medication, changing insulin delivery	31 (1.8)
Stigma	Tweet discusses stigma surrounding anti-diabetic drugs.	Specific situations associated with insulin delivery, reducing stigma, opinions of people without diabetes	29 (1.7)
Dose	Tweet discusses dosing of anti-diabetic drugs. This includes stating the dose, saying how it is taken, or general statements about having to change the dose.	Stating the dose and calculating doses	28 (1.6)
Adverse drug reaction	Tweet is about an experience of an adverse drug reaction. These should be tweets about adverse drug reactions that have actually happened, rather than beliefs about the potential side effects of an anti-diabetic drug.	Specific side effects, general side effects, associated with insulin delivery	21 (1.3)
Abuse	Tweet discusses taking the anti-diabetic drug for nonmedical reasons.	Intent to kill or for fun	10 (0.6)
Nonclassifiable	Some tweets did not provide enough context to determine what it was about.	Too short or incomprehensible	85 (5.1)

#### Anti-Diabetic Drugs Are Too Expensive

The cost of insulin was the most common topic. Some tweeters listed the cost while others described them as “too expensive” (669/1664, 40.2%). Tweeters also remarked that the cost had “skyrocketed.” Health care practitioners were aware that the high cost affected the health of their patients. They described how prices had increased during their time and how they tried to prescribe low-cost anti-diabetic drugs. Cost was an issue for both those with and without health insurance coverage. Certain insurance plans cover certain drugs but not insulin. Younger people expressed fears about aging out of their parents’ insurance.

It was generally felt that high costs were unfair and the profit margin too great. Many believed that anti-diabetic drugs should be free. This was fueled by comparisons of the costs outside the United States or comparisons to other medications. The health consequences of being unable to afford anti-diabetic drugs were extensively discussed. Tweeters expressed difficulty in achieving blood glucose level targets, which they reported resulted in long-term repercussions such as losing limbs, going blind, renal failure, and strokes. Diabetic ketoacidosis was mentioned as a specific concern, and the worst case scenario was death. There were also economic and social consequences such as bankruptcy and homelessness. Some tweeters had made lifestyle decisions based solely on their need for anti-diabetic drugs such as taking a job with insurance rather than a preferred job. Tweeters were open in discussing ways of affording anti-diabetic drugs, including asking other tweeters for money, selling their belongings, or working more than one job. Alternative options were buying cheaper anti-diabetic drugs from abroad, buying over-the-counter medicines, or turning to the black market. Large-scale approaches to making anti-diabetic drugs more affordable included using Twitter to promote campaigns such as the #InsulinForAll movement (a campaign launched in the lead up to World Diabetes Day in 2014 by The Pendsey Trust and T1 International) and to contact people in power, with tweets being sent to the US President and pharmaceutical companies.

#### Anti-Diabetic Drugs Have Varying Efficacy

There was an agreement that insulin was lifesaving. Short-term benefits such as glucose control were noted, as well as generally feeling better. Some tweeters reported issues with their insulin such as insufficient blood glucose reductions, and there were concerns about “Walmart insulin,” with some posts claiming that it is ineffective and caused hypoglycemia. Noninsulin anti-diabetic drugs were perceived to have different levels of efficacy (465/1664, 27.9%). For instance, exenatide and empagliflozin were viewed as effective in reducing weight, which was viewed favorably. Another SGLT2 inhibitor, canagliflozin, was reported to prevent microvascular complications. Metformin had mixed reviews; some felt it worked while others did not.

#### Wealth of Information on Anti-Diabetic Drugs

Information was mostly tweeted as links to or summaries of journal articles (371/1664, 22.2%). Articles varied from laboratory studies to efficacy evaluations. Studies exploring alternative methods of insulin delivery and the use of noninsulin anti-diabetic drugs as adjunct therapies in type 1 diabetes were considered particularly important. Information also came in the form of videos and links to reports on drug approvals and safety published by regulatory bodies.

#### Anti-Diabetic Drugs Are Not Always Available

Problems in availability included delays in mail orders, stolen, or lost medication (158/1664, 9.5%). There were posts calling for wider availability of nonprescription insulin. Some tweeters reported use of nonofficial outlets, and Twitter was used to find, sell, or give away extra supplies. Others discussed anti-diabetic drug availability on a national scale. The main topic concerning the United Kingdom was the impact of leaving the European Union. Additional barriers in the United States were the government shutdown from December 22, 2018 to January 25, 2019 [32], which caused financial and logistic issues, impaired access for deported immigrants, and US sanctions on Venezuela. Tweeters were proactive in discussing ways to ensure their anti-diabetic drug supply, such as stockpiling in the United Kingdom or traveling to Canada or Mexico from the United States. However, there were concerns over stockpiling due to storage issues and insulin’s shelf-life and a strong sense that people should not need to travel abroad to receive life-saving medications.

#### Adherence Can Be Difficult

The majority of tweeters reporting nonadherence mentioned missing doses (124/1664, 7.5%). Those mentioning metformin or liraglutide simply stated they had missed a dose, while insulin users provided more detailed reasons. Some forgot to take their insulin or had equipment problems; others deliberately choose not to take it. Reasons for this included dislike of needles, reactions to news stories condemning insulin, diabulimia with tweeters restricting their insulin intake to control their weight, and incorrectly following advice (this included injecting insulin through clothes or failing to take bolus insulin if not eating due to illness). The most commonly cited reason for nonadherence was cost (85/124, 68.5%), which led to rationing either by taking less insulin per injection or by omitting injections. Some who were not then rationing expressed fears about having to in the future. Insulin overdoses were less commonly discussed, with causes including misreading the dose volume or accidentally taking 2 injections. The only issue reported by tweeters who took an overdose was hypoglycemia.

#### Tweeters Hold a Range of Personal Beliefs

Some Tweeters stated preferences for particular anti-diabetic drugs that had no scientific evidence for the mechanism of action (94/1664, 5.6%). For instance, there was a perception that insulin makes type 2 diabetes worse. Tweeters with diabetes were mostly negative about being on anti-diabetic drugs, expressing that anti-diabetic drugs make life difficult. Some of these negative attitudes centered around equipment, including not liking the “huge” exenatide needles or the hassle of changing cartridges in prefilled insulin pens.

#### Anti-Diabetic Drugs Are Considered Alongside Other Treatments

Anti-diabetic drugs were discussed alongside lifestyle changes, particularly diet changes and specific diets, including the ketogenic diet or a vegan lifestyle (54/1664, 3.2%). Mentions of herbal treatments centered around a news story about the death of a person with type 1 diabetes whose herbalist advised the person to stop his/her insulin. Those using alternative or supplementary treatments were happy to do so, and many expressed annoyance at being offered anti-diabetic drugs with no option of management through lifestyle changes. Subsequently, these alternative treatments were discovered through social media or personal research rather than being initiated by a health care provider. The only alternative treatments that health care providers tweeted support for were exercise and ketogenic diets. Those with type 1 diabetes expressed frustration at being told to try nondrug treatments, particularly diet changes. Although they recognized that reducing carbohydrate intake can reduce insulin requirements, some felt the need to state that type 1 diabetes requires insulin, regardless of diet.

#### Anti-Diabetic Drugs Generate Questions

Those struggling to adjust their anti-diabetic drugs to adequately control their blood glucose levels sought advice from others, and there were questions about where to source “cheap” insulin (41/1664, 2.5%). Health care professionals asked their peers questions, including on the correct anti-diabetic drug, on theoretic scenarios, or interpretation of study findings.

#### Anti-Diabetic Drug Regimens Can Change

Tweeters with type 2 diabetes actively tried to avoid starting insulin. Similarly, stopping insulin was seen as an achievement. Those who had previously managed with only lifestyle changes felt apprehensive about initiating medications. Some tweeters completely stopped their anti-diabetic drugs, usually with guidance from health care providers and changing to a nondrug therapy. Insulin users reported changing to different types of insulin or administration method rather than a different class of anti-diabetic drugs. These data were captured from 1.8% (31/1664) of the tweets.

#### Anti-Diabetic Drugs Are Associated With Stigma

Taking insulin injections in the public resulted in perceptions of being judged or objection to the practice. Those wearing an insulin device or with scars and bruising due to needles felt these drew unwanted attention. Stigma was greater at airport checkpoints, work, or school. These data were captured from 1.7% of the tweets (29/1664). Some tweets discussed a reduction in stigma. This included restaurants providing carbohydrate content information to facilitate insulin dosing and the sense of togetherness when an individual saw other patients with diabetes taking injections. Some tweeters who did not have diabetes believed that there was no stigma for patients with diabetes, arguing that, “patients with diabetes are not judged for using insulin; so, why should people with depression be judged for taking antidepressants?”

#### Dosing Varies Based on the Anti-Diabetic Drug

Dosing based on meal-time carbohydrate or protein intake was noted to be difficult. Some tweeters shared their calculations. Some tweeters admitted to guessing their doses but that was not effective. For tweeters on noninsulin anti-diabetic drugs, doses were decided upon by health care providers. These data were captured from 1.7% of the tweets (28/1664).

#### Anti-Diabetic Drugs Can Cause Adverse Drug Reactions

The explicitness of the descriptions of the adverse drug reactions varied. Gastrointestinal issues, including vomiting or stomach aches, were mentioned for metformin and empagliflozin. Insulin and pioglitazone were both reported to cause weight issues. Other adverse drug reactions included allergic reactions to insulin, cognitive issues with metformin, and blood count changes with empagliflozin. Some adverse reactions were specific to the mode of insulin delivery, including local skin reactions to injections and scar tissue formation following the use of pumps. Other tweeters stated they had an adverse reaction but did not explain further. Tweeters discussed ways to cope, such as by spreading out the doses. The only adverse reaction that seemed to cause cessation was near-death experiences in 3 cases. These data were captured from 1.6% of the tweets (28/1664).

#### Anti-Diabetic Drugs Can Be Abused

There were first-person reports of deliberately taking too much insulin for the thrill of trying to restabilize blood glucose levels. Insulin was recognized as potentially deadly—there were tweets about people trying to kill themselves or someone else by administering insulin. These data were captured from 0.6% of the tweets (10/1664).

### Non–Health-Related Tweets

While this study’s primary focus was the exploration of health-related tweets, it became evident that trends within the non–health-related tweets were also important (1556/1664). Though some non–health-related tweets were jokes or advertisements, 59.1% (920/1556) of the tweets were on the cost of anti-diabetic drugs—these raised similar issues to the health-related cost tweets without discussing the health implications.

## Discussion

### Overview

This study explored public perceptions of anti-diabetic drugs via the analysis of health-related tweets. We found that the issue of cost dominated both health and non–health-related tweets regarding insulin and overwhelmed our results, with implications for other identified themes such as availability, adherence (via rationing), and safety of cheaper versions. We found a similar proportion of health-related tweets in our sample (1664/4000, 41.6%) when compared to that in our study on statins (5201/11,852, 43.8%) [33]. However, the excluded non–health-related tweets differed from those on statins. People tweeting on the non–health-related aspects of anti-diabetic drugs often referred to cost or unfair pricing, while non–health-related tweets on statins were often cultural references, jokes, financial or news reports, or web-based pharmacies.

Within our health-related tweets, it was possible to identify whether the person tweeting was discussing their own diabetes in 24.9% of the cases (415/1664), someone known to them with diabetes in 17.9% of the cases (298/1664), or if they were in a health care profession (45/1664, 2.7%). Interestingly, with those tweeting on statins [33], it was possible to identify whether the person tweeting was taking statins in 32.8% of the cases (1707/5201), someone they know taking statins in 6.6% of the cases (346/5201), or whether the person was a health care professional (325/5201, 6.2%). The much higher proportion of people discussing someone known to them with diabetes may be because of the large scale concern for people with diabetes not being able to afford their insulin.

While type 2 diabetes makes up 90% of the global cases of diabetes [1], for those tweets where we could decipher the type of diabetes more were from people with type 1 than from people with type 2 diabetes and in line with this, insulin was by far the most discussed drug (9107/9793, 92.9% of the tweets). When considering that 44.7% of the people with type 1 diabetes are younger than 40 years compared to just 4% of the people with type 2 diabetes [34] and two-thirds of Twitter users are younger than 35 years [35], a possible partial explanation is that the Twitter demographic is more aligned with the younger demographic with type 1 diabetes. Another explanation is the high proportion of people discussing the injustice of the high cost of insulin for type 1 diabetes.

The implications of high-cost insulin were far reaching. While tweets reporting bankruptcy, stealing, and homelessness associated with the cost of insulin may seem like extreme subjects to post on a public platform, a study in 2020 with individuals with type 1 diabetes in the United States corroborated these stories [36]. Approximately 39.2% of the patients struggling to afford their insulin do not tell their health care professionals [37], making Twitter a potential way of identifying patients in need. Tweets about the increasing cost of insulin reflect the general trend in the United States. The price of insulin glargine—the most commonly prescribed insulin in the United States [38]—increased by 117% over 7 years [39]. Even for those who have a Medicare insurance plan, diabetes-related out-of-pocket spending increased by 10% per year between 2006 and 2013 [40]. This is despite the average spending for other prescription medications only increasing by 2.8% over the same period [40]. An analysis of the tweets about statins found that only 3.5% (182/5201) of the tweets mentioned cost [33] compared to 40.2% (669/1664) of the tweets in this study. This may be because the cost of a month’s supply of statins, on average, is only one-third of the price of a month’s supply of anti-diabetic drugs [41].

A relationship between cost and availability, adherence, safety and efficacy was apparent from the tweets. Twitter appeared to be an informal marketplace for trading anti-diabetic drugs, although we did not confirm actual transactions. The overall sentiment of the tweets is that the lack of affordable anti-diabetic drugs is unfair and detrimental to health, which is in agreement with the findings of Litchman et al [42], who reported that those giving away their extra anti-diabetic drugs did so out of altruism and frustration at the lack of pricing regulations rather than the need to profit. Some tweeters travelled abroad to purchase their anti-diabetic drugs; these tweeters are among the estimated 2.3 million US individuals who buy their medications abroad [43]. Although this analysis cannot quantify how many individuals do this, it does give an insight into the reasons specific to anti-diabetic drugs. Prior research has found that those without health insurance are most likely to purchase prescription medications abroad [43], and this was reflected in the tweets. Of note, Hong et al [43] inferred that those seeking health information on the internet or using web-based chat groups were twice as likely to purchase medications abroad; therefore, given that this is a Twitter analysis, there may be an overrepresentation of individuals who purchase their anti-diabetic drugs in this way. It is currently illegal to purchase insulin abroad and import it into the United States for personal use [44]; therefore, the fear of being caught may explain why there has been little mention of this method in previous studies. In July 2019, the Food and Drug Administration proposed the Safe Importation Action Plan, intending to facilitate the import of medications from Canada [45]. Despite the tweet collection covering this period, there were no tweets related to this, questioning how far this announcement spread. The tweet collection period coincided with several delays to the date the United Kingdom was due to leave the European Union. Tweets related to this highlighted the importance of protecting medication imports. The worries about imports are supported by Holt et al [46], who noted that only animal insulin is manufactured in the United Kingdom, with Novo Nordisk, Eli Lilly, and Sanofi having to import their insulins.

This study indicates the potential impact of high-cost insulin and concerns about availability, leading to rationing. This in line with the results of a global survey of 1478 individuals with type 1 diabetes, and their care providers reported that 25.9% of the respondents from the United States had rationed their insulin at some point in the last year [47]. Rationing is deeply problematic and there was a little debate regarding insulin’s effectiveness, with powerful descriptions of how it is lifesaving. Participants with type 1 diabetes in a previous study described insulin as “life or death” for them [36], but this analysis shows that the general public also appreciates the life-saving nature of insulin. We found little evidence of the stigma associated with being on insulin among people with type 1 diabetes, which has been reported in previous studies [48]. The growing empathy for people with type 1 diabetes because of the high prices of insulin may be interconnected with a decline in the stigma.

Opinions on the efficacy of anti-diabetic drugs to treat type 2 diabetes were more varied; many tweeters expressed their desire to stop their medication, and tweets discussing other treatment options for type 2 diabetes seemed to favor dietary changes. Other studies have also indicated poor adherence in type 2 diabetes [49]. With respect to type 2 diabetes, people experience more stigma when on insulin than when on a noninsulin anti-diabetic drug [50]. A qualitative systematic review found that health care providers often doubt their patients’ ability to self-manage their diabetes, consequently preferring a paternalistic approach [51]. This is reflected in the sense of annoyance among the tweeters at not being given the option to manage type 2 diabetes by lifestyle changes alone.

There has been interest in using Twitter as a source for collecting anecdotal accounts of adverse drug reactions [13]. In our analysis of statins [33], we identified 6.8% (353/5201) of the tweets to be about adverse reactions compared to just 1.3% (21/1664) in this study. This was unexpected, given that dose-related serious adverse effects with drugs to treat diabetes are considered to be among the adverse drug effects with the highest public health impact [52], while statins have a much higher degree of safety. The cheap version ReliOn (Walmart insulin) was the only type of insulin that had its efficacy and safety questioned.

A major source of criticism of social media is the high volume of misinformation. Misinformation on social media can have detrimental effects on health behaviors, and they are difficult to correct once they gain acceptance [53]. We found little evidence of misinformation among our tweets, and in line with the literature, no misinformation was shared by health care professionals [53]. Broadly, there were 2 ways individuals used Twitter to discuss anti-diabetic drugs. The first was as a microblogging site for recording day-to-day experiences such as trying to afford their insulin, rationing, side effects, and incidences involving stigma. These tweets may provide a useful introduction into what life is like while taking anti-diabetic drugs, which could influence the support provided by health care professionals. Alternatively, Twitter was used as a tool that was intended to bring about change, with tweeters discussing complex social issues. This is pertinent to policymakers as it highlights the issues that both patients and the public consider most pressing.

### Strengths and Limitations

The large volume of Twitter data from a mix of tweeters with and without diabetes allowed an insight into a broad range of perspectives. Manual coding was used during the tweet analysis, which is considered the gold standard method [28]. While the use of automated computer programs may be quicker and can allow large data sets to be coded, they are associated with lower accuracy [22]. These findings represent the perspectives of the Twitter-using population but not necessarily the general population [54]. As an illustration, in the United States, the average tweeter is likely to be White, young, well-educated, and a Democrat [54]. As this study did not collect demographic data, it is hard to appreciate which population this study does reflect. Since Twitter is available worldwide, this study planned to take a global approach to anti-diabetic drug perceptions, but upon analysis, it became evident that a large burden of the tweets centered around issues in the United States. It was only after the research process began that Patel et al [55] published their analysis of 50,286 diabetes-related tweets, indicating that 43.6% of the tweets came from the United States, followed by 14.9% from the United Kingdom. Despite the large volume of tweets, we only identified issues relevant to a few countries and were unable to compare differences among countries, as we did not collect the geolocations of the Twitter users. Future work could address this. The limited non-US issues collected may, in part, be because of the search terms we used and that we only used a single social media platform. Other platforms may be needed to explore perceptions from a wider population and in other countries. Our analysis does not go beyond content analysis. We did not record any user engagement metrics or interactions. We were also unable to verify any of the claims made, and people may post things on the internet that they would not say in person. However, the fact that information shared on social media is expressed spontaneously in an open digital space with a flat role hierarchy is a major advantage for capturing perceptions that otherwise would not be reported [56]. Finally, we were unable to distinguish whether posts were referring to type 1 or type 2 diabetes in the majority of the tweets. Issues with anti-diabetic drugs are likely to be dependent on the type of diabetes. This limitation may be generalizable to other medications studied on social media, which are used for more than one indication.

### Conclusion

The use of Twitter has provided an insight into the immediate perceptions of anti-diabetic drugs outside of a clinical setting, thereby giving a unique perspective. Not only does this study support the findings already established in the current literature, but it has also provided an appreciation of the struggles of people taking anti-diabetic drugs, particularly in light of the high cost of insulin. This study has also shown that the public is aware of these issues and are waiting for governments and health care systems to make changes.

## Appendix

Multimedia Appendix 1 Key terms used for the search.
