# Supplementary material for: Public Perspectives on Anti-Diabetic Drugs: Exploratory Analysis of Twitter Posts
Source: JMIR Diabetes. 2021 Jan 26;6(1):e24681. doi: 10.2196/24681 (PMC7872831; doi:10.2196/24681)
Supplement: Multimedia Appendix 1 [file diabetes_v6i1e24681_app1.docx]

**Textbox 1.** Key terms selected by the University of Pennsylvania to prime to the application programming interface.

| - Actos - Albiglutide - Alogliptin - Amaryl - Apidra - Aspart - Avandia - Basaglar - Bydureon - Byetta - Canagliflozin - Chlorpropamide - Dapagliflozin - Degludec - Determir - Diabeta - Diabetes… - Diacetate - Dulaglutide - Empagliflozin - Ertugliflozin - Ertugliflozin - Exenatide - Farxiga - Fiasp - FlexPen - Formamet - Glargine - Gliclazide - Glimepiride - Glipizide - Glucophage - Glucotrol - Glulisine - Glumetza - GlynaseGylburide - Humalog - Humolog - Humulin - Humulog - Insilin - Insulan - Insulin - Insuling - Insulins - Insullin - Insuln - Invokana - Isophane - Janumet - Januvia - Jardiance - Kanazo - Lantas - Lantis - Lantus - Levemir - Linagliptin - Liraglutide - Lispro - Metaformin - Metaglip - Metform - Metforman - Metformin - Metformine - Micronase - Micronized - Miglitol - Nateglinide - Nesina Novalog - Novolin - Novolog - Onglyza - Orinase - Oseni - Ozempic - Pioglitazone - Pramlintide - Prandin - Repaglinide - Repaglinide - Riomet - Rosiglitazone - Ryzodeg - Ryzodeg - Saxagliptin - Semaglutide - Sitagliptin - Starlix - Steglatro - Tol-Tab - Tolazamide - Tolbutamide - Tolinase - Toujeo - Tradename - Tradjenta - Trajenta - Trandate - Tresiba - Trulicity - Victoza - Vildagliptin - Zonulin |
| --- |
